# Supplementary material for: Astrocyte GluN2C NMDA receptors control basal synaptic strengths of hippocampal CA1 pyramidal neurons in the stratum radiatum
Source: eLife. 2021 Oct 25;10:e70818. doi: 10.7554/eLife.70818 (PMC8594917; doi:10.7554/eLife.70818)
Supplement: Transparent reporting form [file elife-70818-transrepform1.docx]

***eLife’s* transparent reporting form**

We encourage authors to provide detailed information *within their submission* to facilitate the interpretation and replication of experiments. Authors can upload supporting documentation to indicate the use of appropriate reporting guidelines for health-related research (see [EQUATOR Network](http://www.equator-network.org/%20)), life science research (see the [BioSharing Information Resource](https://biosharing.org/)), or the [ARRIVE guidelines](http://www.plosbiology.org/article/info:doi/10.1371/journal.pbio.1000412) for reporting work involving animal research. Where applicable, authors should refer to any relevant reporting standards documents in this form.

If you have any questions, please consult our Journal Policies and/or contact us: [editorial@elifesciences.org](mailto:editorial@elifesciences.org).

**Sample-size estimation**

- You should state whether an appropriate sample size was computed when the study was being designed
- You should state the statistical method of sample size computation and any required assumptions
- If no explicit power analysis was used, you should describe how you decided what sample (replicate) size (number) to use

Please outline where this information can be found within the submission (e.g., sections or figure legends), or explain why this information doesn’t apply to your submission:

Sample sizes for electrophysiology experiments were determined based on PPR disparity obtained in pilot studies and experimental data outlined in Letellier et al., 2016 PNAS. A power analysis was performed using a $\beta$ value of 0.8 and an $\alpha$ value of 0.05. Standard deviations for PPR disparity data were determined to be ~0.35, and expected effect size is ~45%.

**Replicates**

- You should report how often each experiment was performed
- You should include a definition of biological versus technical replication
- The data obtained should be provided and sufficient information should be provided to indicate the number of independent biological and/or technical replicates
- If you encountered any outliers, you should describe how these were handled
- Criteria for exclusion/inclusion of data should be clearly stated
- High-throughput sequence data should be uploaded before submission, with a private link for reviewers provided (these are available from both GEO and ArrayExpress)

Please outline where this information can be found within the submission (e.g., sections or figure legends), or explain why this information doesn’t apply to your submission:

Each electrophysiology experiment was repeated in accordance to the sample sizes established by the power analysis. For PPR distributions, a single replicate is considered a single synaptic input, sampled from a single cell, in a single brain slice. An input is defined as a small bundle of axons that independently contributes to the generation of a monosynaptic excitatory postsynaptic potential (EPSC) in the recorded cell. Up to three separate inputs were activated per recorded cell, per slice, and multiple slices were obtained from single animals.

Exclusion of data was based on the quality of the patch recording. Only recordings in which low and stable access resistance was achieved were analysed.

The above information is summarized in the methods section under the heading ‘Slice preparation and whole cell recordings’, and ‘Recordings of synaptic transmission’. Additional information is contained in the Ephys Statistics Report_rev.

**Statistical reporting**

- Statistical analysis methods should be described and justified
- Raw data should be presented in figures whenever informative to do so (typically when N per group is less than 10)
- For each experiment, you should identify the statistical tests used, exact values of N, definitions of center, methods of multiple test correction, and dispersion and precision measures (e.g., mean, median, SD, SEM, confidence intervals; and, for the major substantive results, a measure of effect size (e.g., Pearson's r, Cohen's d)
- Report exact p-values wherever possible alongside the summary statistics and 95% confidence intervals. These should be reported for all key questions and not only when the p-value is less than 0.05.

Please outline where this information can be found within the submission (e.g., sections or figure legends), or explain why this information doesn’t apply to your submission:

This information is contained in Figures 1, 2, 6, 7 and respective figure legends. Additional information is contained in Ephys Statistics Report_rev.

(For large datasets, or papers with a very large number of statistical tests, you may upload a single table file with tests, Ns, etc., with reference to sections in the manuscript.)

**Group allocation**

- Indicate how samples were allocated into experimental groups (in the case of clinical studies, please specify allocation to treatment method); if randomization was used, please also state if restricted randomization was applied
- Indicate if masking was used during group allocation, data collection and/or data analysis

Please outline where this information can be found within the submission (e.g., sections or figure legends), or explain why this information doesn’t apply to your submission:

Group allocation was randomized. Restricted randomization was not applied.

**Additional data files (“source data”)**

- We encourage you to upload relevant additional data files, such as numerical data that are represented as a graph in a figure, or as a summary table
- Where provided, these should be in the most useful format, and they can be uploaded as “Source data” files linked to a main figure or table
- Include model definition files including the full list of parameters used
- Include code used for data analysis (e.g., R, MatLab)
- Avoid stating that data files are “available upon request”

A summary of all electrophysiology experimental data is contained in the attached Ephys Statistics Report_rev.

Mathematical models shown in Figure 3 and Figure 4 are generated by the model programmed in Python. The source codes are included in the attached file.

Please indicate the figures or tables for which source data files have been provided:
